# The P2Y_2_ Receptor Interacts with VE-Cadherin and VEGF Receptor-2 to Regulate Rac1 Activity in Endothelial Cells

**DOI:** 10.4236/jbise.2014.714109

**Published:** 2014-12-01

**Authors:** Zhongji Liao, Chen Cao, Jianjie Wang, Virginia H. Huxley, Olga Baker, Gary A. Weisman, Laurie Erb

**Affiliations:** 1 Department of Medicine, University of California, San Diego, USA; 2 Department of Biochemistry, Life Sciences Center, University of Missouri, Columbia, USA; 3 Department of Biomedical Sciences, Missouri State University, Springfield, USA; 4 Department of Medical Pharmacology and Physiology, University of Missouri, Columbia, USA; 5 School of Dentistry, University of Utah, Salt Lake City, USA

**Keywords:** VE-Cadherin, P2Y Receptors, Rac, Endothelium, Adherens Junctions

## Abstract

Vascular endothelial cadherin (VE-cadherin) mediates homophylic adhesion between endothelial cells and is an important regulator of angiogenesis, blood vessel permeability and leukocyte trafficking. Rac1, a member of the Rho family of GTPases, controls VE-cadherin adhesion by acting downstream of several growth factors, including angiopoietin-1 and vascular endothelial growth factor (VEGF). Here we show that UTP-induced activation of the G_q_ protein-coupled P2Y_2_ nucleotide receptor (P2Y_2_R) in human coronary artery endothelial cells (HCAECs) activated Rac1 and caused a transient complex to form between P2Y_2_R, VE-cadherin and VEGF receptor-2 (VEGFR-2). Knockdown of VE-cadherin expression with siRNA did not affect UTP-induced activation of extracellular signal-regulated kinases 1/2 (ERK1/2) but led to a loss of UTP-induced Rac1 activation and tyrosine phosphorylation of p120 catenin, a cytoplasmic protein known to interact with VE-cadherin. Activation of the P2Y_2_R by UTP also caused a prolonged interaction between p120 catenin and vav2 (a guanine nucleotide exchange factor for Rac) that correlated with the kinetics of UTP-induced tyrosine phosphorylation of p120 catenin and VE-cadherin. Inhibitors of VEGFR-2 (SU1498) or Src (PP2) significantly diminished UTP-induced Rac1 activation, tyrosine phosphorylation of p120 catenin and VE-cadherin, and association of the P2Y_2_R with VE-cadherin and p120 catenin with vav2. These findings suggest that the P2Y_2_R uses Src and VEGFR-2 to mediate association of the P2Y_2_R with VE-cadherin complexes in endothelial adherens junctions to activate Rac1.

## 1. Introduction

The vascular endothelium separates circulating blood from the underlying tissue and provides a semipermeable barrier for normal fluid, nutrient and waste exchange. During inflammatory and allergic reactions, however, the endothelial barrier is disrupted causing tissue swelling (edema) that is often accompanied by leukocyte infiltration. It is well known that the integrity of the endothelial barrier is controlled by the formation and disruption of intercellular adhesion structures comprised of adherens junctions and tight junctions and also by contractility provided by the actomyosin cytoskeleton [1]. The Rho family of small GTPases (RhoA, Rac1 and Cdc42) regulates cytoskeletal organization and the stability of intercellular junctions [2] and many inflammatory mediators, such as vascular endothelial growth factor (VEGF), lipopolysaccharide (LPS), thrombin, tumor necrosis factor α (TNFα), bradykinin, histamine and also leukocytes, modulate endothelial permeability by altering the activities of Rho GTPases [3]. Over the past decade, it has become apparent that Rho GTPases can have both stabilizing and destabilizing effects on the endothelial barrier depending on their context within the cell as well as through complex interactions with regulators of G protein signaling (guanine nucleotide exchange factors, GEFs; guanine dissociation inhibitors, GDIs; and GTPase accelerating proteins, GAPs) [3] [4].

Recently, the P2Y_2_ nucleotide receptor (P2Y_2_R), a G_q_ protein-coupled receptor activated equally well by ATP and UTP, has emerged as an important regulator of blood vessel permeability and leukocyte recruitment. *In vivo* studies focusing on the P2Y_2_R have shown that activation of this receptor transiently increases microvascular leakage to macromolecules [5] and promotes extravasation of leukocytes in inflammatory conditions involving both micro- and macrovessels, including atherosclerosis, asthmatic airway inflammation, Alzheimer’s disease, autoimmune diseases, bacterial infection and chronic obstructive pulmonary disease (COPD) [6]–[11]. In addition, a role for the P2Y_2_R in cancer metastasis was recently demonstrated by Schumacher *et al.* [12], who showed that platelets activated by tumor cells release ATP, which promotes transendothelial migration and metastasis of tumor cells through activation of the P2Y_2_R. Other studies demonstrated that the P2Y_2_R, by virtue of an arginine-glycine-aspartate (RGD) integrin-binding motif in its extracellular domain, mediates the activation of small Rho GTPases, Rac1 and RhoA [13] [14] and, by virtue of SH3-binding motifs in its intracellular domain, interacts with Src and promotes the Src-dependent activation of several growth factor receptors, including VEGFR-2 that up-regulates the expression of vascular cell adhesion molecule-1 (VCAM-1), a leukocyte binding protein in endothelial cells [15] [16]. Since the P2Y_2_R regulates vascular integrity, leukocyte adhesion and extravasation, and Rho GTPase activities [5]–[11] [13]–[17], we speculated that the P2Y_2_R may modulate the permeability of endothelium by affecting the stability of adherens junctions.

Among the proteins in endothelial cell junctions, vascular endothelial cadherin (VE-cadherin) is well recognized for its role in regulating vascular permeability and leukocyte extravasation [18]–[21]. VE-cadherin is exclusively expressed in vascular endothelial cells [22] and deletion of VE-cadherin in mice causes severe defects in vascular development and embryonic death [23] [24]. Down-regulation of VE-cadherin has been associated with vascular tumor growth [25], whereas treatment of endothelial cells with VE-cadherin neutralizing antibody increases VEGF-induced VEGFR-2 activity [26]. Compared to endothelial cells expressing VE-cadherin, VE-cadherin-null endothelial cells have thinner actin stress fibers, less vinculin-positive focal contacts, and lower activity of Rac1 [27]. The N-terminal extracellular domain of VE-cadherin mediates Ca^2+^-dependent homophilic adhesion while the cytoplasmic domain interacts with various intracellular binding partners, including p120 and β-/γ-catenins, the latter of which may provide a linkage to the actin cytoskeleton through interaction with α-catenin [28]. Modulation of cell-cell contacts that regulate cell adhesion and cell motility likely requires interactions between cadherins and catenins, and it has been shown that p120 catenin regulates actin cytoskeletal organization and cell motility by activation of Rho GTPases [29]–[31]. In addition, VE-cadherin associates with VEGFR-2, intracellular signaling molecules, such as Shc and Csk [32] [33], and vascular endothelial protein tyrosine phosphatase (VE-PTP) [34]. These interactions are thought to be important for regulating cell-cell contacts, cell adhesion, and growth factor signaling [22].

In the present study, we investigated how activation of the P2Y_2_R in human coronary artery endothelial cells (HCAECs) affects receptor distribution and association with VE-cadherin. Our previous work demonstrated that activation of the P2Y_2_R promotes monocyte adhesion and extravasation into rabbit carotid arteries and increases the development of atherosclerotic plaques [6]. Furthermore, mechanistic studies in HCAECs revealed that the activated P2Y_2_R associates transiently with VEGFR-2 in a Src-dependent manner and that VEGFR-2 activity is necessary for VCAM-1 up-regulation induced by UTP [16]. Here, we demonstrate that activation of the P2Y_2_R in HCAECs causes translocation of this receptor to intercellular junctions and transient association with VE-cadherin. We also show that Rac1 activation by the P2Y_2_R in HCAECs involves tyrosine phosphorylation of VE-cadherin and p120 catenin and association of p120 catenin with vav2, a GEF for Rac1.

## 2. Materials and Methods

### 2.1. Materials

Goat anti-human VE-cadherin polyclonal antibody, rabbit anti-human Flk-1 (VEGFR-2) polyclonal antibody, and rabbit anti-vav2 polyclonal antibody were purchased from Santa Cruz Biotechnology (Santa Cruz, CA). The mouse anti-phosphotyrosine antibody was purchased from BD Bioscience (San Jose, CA). Mouse anti-HA antibody conjugated agarose beads and rabbit anti-HA antibody were purchased from Covance (Berkeley, CA). Anti-p120 catenin antibodies were purchased from Santa Cruz Biotechnology and BD Bioscience. Rabbit polyclonal anti-phospho-ERK1/2 (extracellular signal-regulated kinases 1/2) antibody was purchased from Cell Signaling (Beverly, MA). Specific inhibitors for VEGFR-2 tyrosine phosphorylation (SU1498) and Src (PP2) were obtained from Calbiochem (Indianapolis, IN). ON-TARGET plus SMART pool siRNA duplexes targeting the human P2Y_2_ receptor, VE-cadherin and p120 were purchased from Dharmacon (Chicago, IL). VE-cadherin cDNA was a kind gift from Dr. Elisabetta Dejana (IFOM-IEO, Milan, Italy). All other reagents including nucleotides were obtained from Sigma-Aldrich (St. Louis, MO), unless otherwise specified.

### 2.2. Cell Culture and Transfection

HCAECs (human coronary artery endothelial cells) were cultured in endothelial basal medium-2 (EBM-2; Clonetics, Walkerville, MD) at 37°C in a humidified atmosphere of 5% CO_2_ and 95% air. HCAECs were used between the fourth and eighth passages. For transient transfections, siRNA or plasmid constructs were delivered using Targefect F-2 plus Virofect or Targefect-HUVEC from Targeting Systems (Santee, CA), respectively, according to the manufacturer’s instructions. In both cases, transfection efficiency of at least 60% was achieved. Human P2Y_2_R (hP2Y_2_R) cDNA encoding a hemagglutinin (HA) tag at the N-terminus in pcDNA3.1(−) [13] or cDNA encoding the hP2Y_2_R cDNA with an enhanced green fluorescent protein (eGFP) tag at the C-terminus in pEGFP-N1 (a kind gift from Dr. Fernando A. González, Department of Chemistry, University of Puerto Rico) was transiently expressed in HCAECs. Human 1321N1 astrocytoma cells lacking endogenous P2 receptors were also used in supplemental experiments. The cells were cultured in Dulbecco’s modified Eagle’s medium (DMEM; Life Technologies, Carlsbad, CA) containing 5% (v/v) fetal bovine serum (FBS), 100 U/ml penicillin and 100 µg/ml streptomycin and maintained at 37°C in a humidified atmosphere of 5% CO_2_ and 95% air. Cells were stably transfected with cDNA encoding either the wild type P2Y_2_R or a mutant P2Y_2_R in which the C-terminal proline-rich SH3-binding domains where deleted (Del), as previously described [15]. These receptor constructs contained sequence encoding a HA tag at the N-terminus of the P2Y_2_R, as previously described [15]. In addition, the cells were transiently transfected with either pcDNA3 vector or pcDNA-VE-cadherin using the Lipofectamine 2000 reagent (Life Technologies, Carlsbad, CA).

### 2.3. Confocal Laser Scanning Microscopy Visualization

HCAECs plated on glass coverslips were cultured to ~90% confluence before being transfected with hP2Y_2_R-eGFP cDNA. The cell transfectants were maintained in growth medium for 72 h and transferred to serum-free medium for 12 h. Then, cells were incubated with or without 100 µM UTP for 5 min at 37°C, washed in ice-cold PBS, fixed for 10 min in 4% (w/v) paraformaldehyde, treated with 0.1% (v/v) Triton X-100 for 5 min, and rinsed in PBS. Fixed cells were incubated with mouse anti-human VE-cadherin antibody (1:100 dilution, BD Bioscience, CA) for 1 h, washed and stained with Alexa Fluor 594 goat anti-mouse IgG (1:200 dilution, Invitrogen) for 1 h. Coverslips were mounted on glass slides in ProLong antifade reagent (Life Technologies, Grand Island, NY) and examined using a Zeiss inverted LSM 510 META confocal laser scanning microscope (CLSM) equipped with a C Apochromat 40× objective. Images were acquired, processed and analyzed with a Zeiss LSM Image Examiner.

### 2.4. RNA Extraction and RT-PCR

Isolation of RNA, cDNA synthesis, and RT-PCR were performed as previously described [35]. Amplification of P2Y_2_R cDNA was performed by RT-PCR using the following oligonucleotide primers: sense 5’-CTTCAACGAGGACTTCAAGTACGTGC-3’, and antisense 5’-CATGTTGATGGCGTTGAGGGTGTGG-3’. Primers for amplification of human G3PDH cDNA were: sense 5’-TGAAGGTCGGAGTCAACGGATTTGGT-3’, and antisense 5’-CATGTGGGCCATGAGGTCCACCAC-3’. Thirty-five amplification cycles were used, with annealing temperatures of 60°C as previously described [35]. PCR products were resolved by 2% (w/v) agarose gel electrophoresis.

### 2.5. Rac1 and RhoA Activity Assays

A Rac1 activation assay kit (EMD Millipore, Billerica, MA) was used to assess Rac1 activity according to the manufacturer’s instructions. Briefly, cells were cultured in 6-well tissue culture dishes in EBM-2 with all supplements and then transferred to serum-free medium for 12 h before incubation with or without UTP for 5 min at 37°C. Then, cells were washed three times with ice-cold PBS, suspended in Lysis Buffer containing 125 mM HEPES, pH 7.5, 750 mM NaCl, 5% (v/v) Igepal CA-630, 50 mM MgCl_2_, 5 mM EDTA and 10% (v/v) glycerol, and the lysates were transferred to 1.5 ml tubes. Thirty microliters of agarose-conjugated p21 binding domain of p21 activated kinase-1 (PAK-1) that only recognizes GTP-bound, *i.e.*, activated Rac1 were added to 500 µl of lysate for 1 h at 4°C. The beads were collected by centrifugation and washed three times with Lysis Buffer. Finally, the beads were resuspended in 40 ml of 2× Laemmli sample buffer (120 mM Tris-HCl, pH 6.8, 2% (w/v) SDS, 10% (w/v) sucrose, 1 mM EDTA, 50 mM dithiothreitol and 0.003% (w/v) Bromophenol Blue) and Western blot analysis (see below) was performed with a 1:1000 dilution of mouse anti-human Rac1 antibody (EMD Millipore, Billerica, MA). RhoA activity was determined similarly, except that p21-PAK-1-agarose and anti-Rac1 antibody were replaced with Rhotekin Rho binding domain (RBD)-agarose and mouse anti-human RhoA antibody (EMD Millipore, Billerica, MA), respectively.

### 2.6. Immunoprecipitation and Immunoblotting

Immunoprecipitation (IP) and immunoblotting (IB) were performed, as previously described [13]. Cells were transferred to serum-free medium for 12 h before treatment with the indicated inhibitors and/or UTP at 37°C and cell lysates were used for IP with the indicated antibodies. The immune complexes were precipitated with protein A- or protein G-conjugated beads and analyzed by IB with antibodies against the proteins of interest. After IB, the membranes were stripped and reprobed with the same antibody used for IP to verify the consistency of protein precipitation between samples. IP was also performed with lysates from cells expressing the HA-tagged hP2Y_2_R using anti-HA-conjugated agarose beads.

### 2.7. Internalization of HA-hP2Y_2_R

Internalization of the HA-hP2Y_2_R was examined indirectly by determining the uptake of HA antibodies added to 1321N1 astrocytoma cells stably expressing the wild type HA-hP2Y_2_R, as described [36]. Briefly, the cells were transfected with either pcDNA3 or pcDNA3-VE-cadherin. Then, cell transfectants were incubated at 37°C in serum-free medium supplemented with 5 µg/ml anti-HA antibodies in the absence or presence of 1 mM UTP for 5 min to allow endocytosis of anti-HA antibody bound to the HA-hP2Y_2_R. Cells were then placed on ice to prevent further receptor internalization, washed with ice-cold PBS, and surface-bound antibodies were removed by three washes with ice-cold acidic buffer (100 mM glycine, 20 mM magnesium acetate, 50 mM potassium chloride, pH 2.2). After an additional wash with ice-cold PBS, the cells were lysed with 2× Laemmli sample buffer, and lysates were analyzed for anti-HA antibodies by immunoblotting. Anti-HA antibodies were detected by chemiluminescence using horseradish peroxidase-conjugated antibodies (1:1000 dilution).

## 3. Results

### 3.1. UTP Causes Clustering of the P2Y_2_R in Endothelial Intercellular Junctions

**Figure 1:**
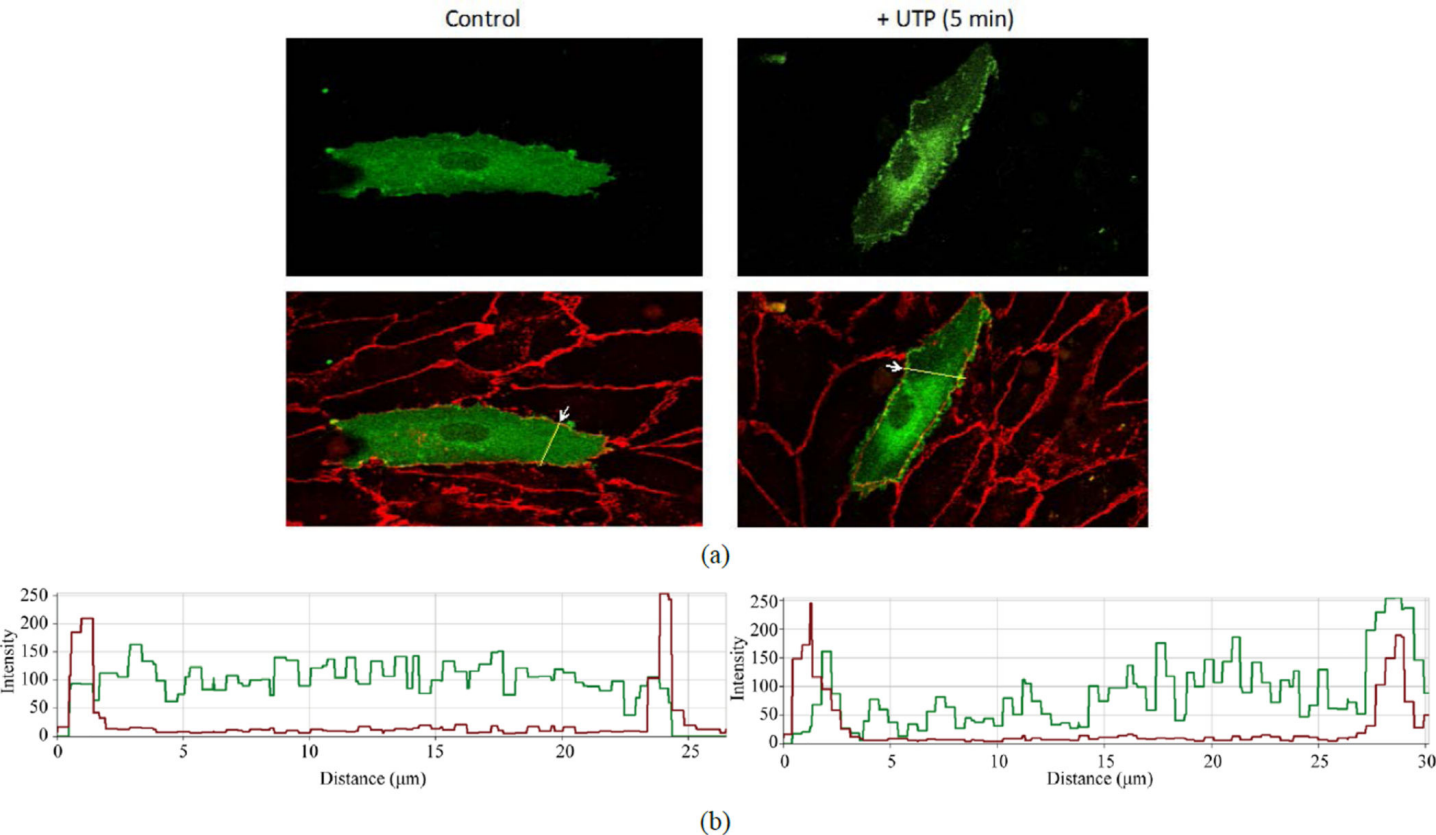
Translocation of eGFP-P2Y2R to peripheral membranes of endothelial cells in response to UTP. (a) HCAECs expressing eGFP-tagged human P2Y2R (shown in green) were stimulated with or without 100 µM UTP for 5 min, as indicated. Cells were washed, fixed, permeabilized and labeled with mouse anti-VE-cadherin antibody. Alexa Fluor 594-conjugated antimouse IgG was used to stain VE-cadherin (shown in red on the bottom pictures); (b) Graphs showing the distribution intensity of VE-cadherin (red line) and eGFP-P2Y2R (green line) across the dual-labeled cells. The yellow lines, indicated by the arrows in (a), show where the distribution graphs were generated. For each treatment, ~30 cell transfectants from 4 independent experiments were examined.

To visualize the distribution of P2Y_2_ receptors in endothelial cell, we transfected HCAECs with cDNA encoding the eGFP-tagged hP2Y_2_R. Thirty-six hours after transfection, most eGFP-hP2Y_2_Rs were localized to an intracellular compartment (not shown), possibly early endosomes, whereas 84 h after transfection, the GFP-hP2Y_2_R appeared uniformly distributed in the plasma membrane (Figure 1). In ~50% of the transfected cells, a 5 min stimulation with UTP caused clustering of eGFP-hP2Y_2_R in peripheral membranes between adjacent cells (Figure 1). The cell peripheries were labeled with an antibody against VE-cadherin, an adherens junction protein involved in endothelial cell-cell adhesion.

### 3.2. UTP Induces Transient Association of VE-Cadherin with P2Y_2_R and VEGFR-2

**Figure 2:**
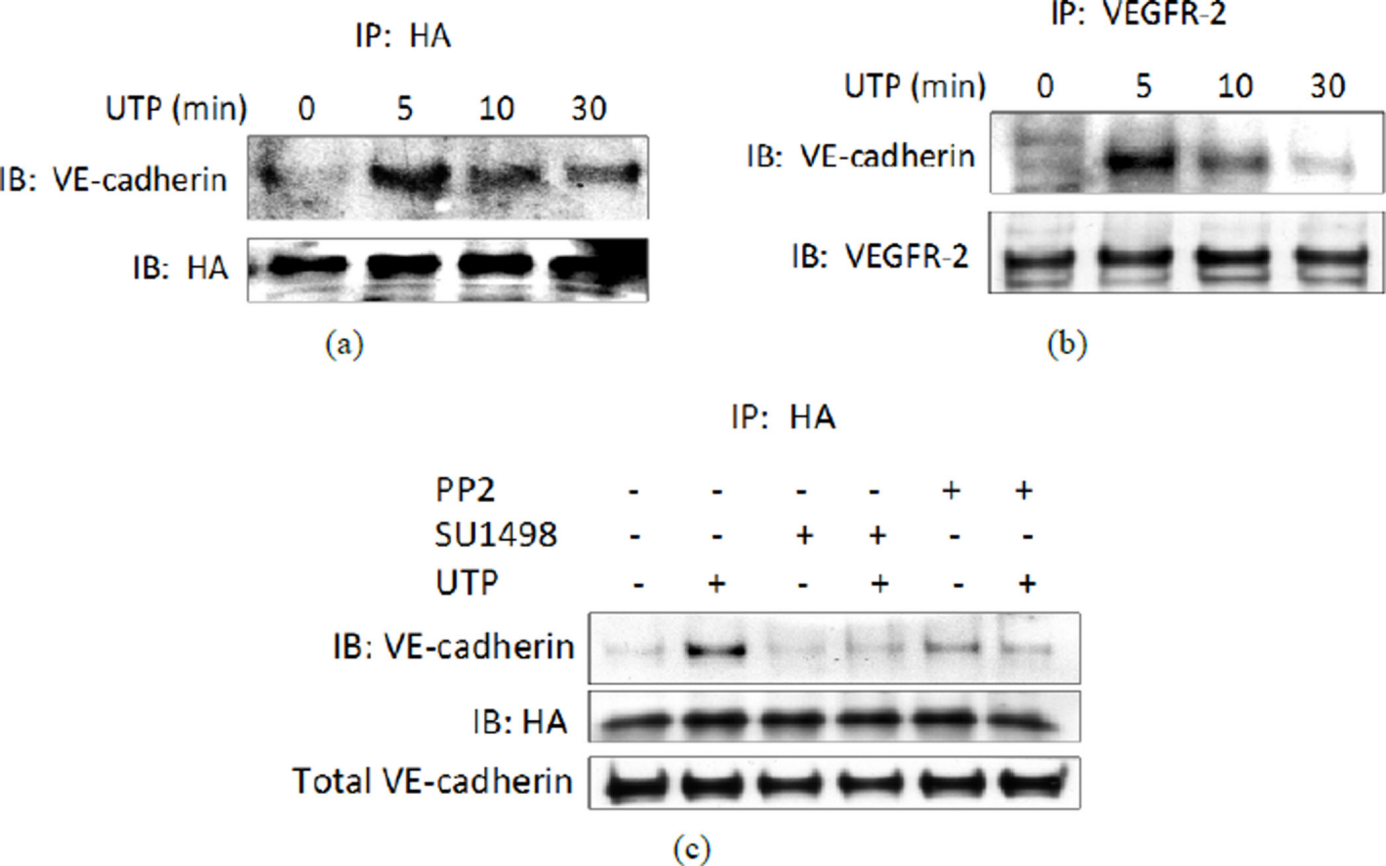
UTP causes transient interaction between the HA-P2Y2R, VE-cadherin and VEGFR-2. (a) HCAECs expressing HA-tagged human P2Y2R were treated with 100 µM UTP for the indicated time. Cell lysates were prepared and subjected to IP with mouse anti-HA antibody conjugated to agarose beads. IB analysis of the IP samples was then performed with anti-VE-cadherin antibody to detect co-precipitation of VE-cadherin or with rabbit anti-HA antibody to detect relative levels of HA-tagged P2Y2R precipitated in each sample; (b) HCAECs were treated with 100 µM UTP for the indicated time and cell lysates were subjected to IP with anti-VEGFR-2 antibody and IB with anti-VE-cadherin antibody. The membrane was stripped and re-blotted with anti-VEGFR-2 antibody; (c) HCAECs expressing HA-tagged human P2Y2R were treated with 1 µM of the Src kinase inhibitor, PP2, or 10 µM of the VEGFR-2 kinase inhibitor, SU1498, for 30 min followed by incubation with or without 100 µM UTP for 5 min. Cell lysates were prepared and subjected to IP with anti-HA matrix beads and IB with anti-VE-cadherin or anti-HA antibody. Cell lysates also were subjected to IB with anti-VE-cadherin antibody to detect total VE-cadherin. Blots representative of 4 experiments are shown.

Since the P2Y_2_R translocated to endothelial intercellular junctions upon activation (Figure 1), we examined whether the activated P2Y_2_R interacts with an adherens junction protein. Accordingly, HCAECs were transfected with cDNA encoding the HA-hP2Y_2_R and IP was performed with anti-HA-conjugated agarose beads. An interaction between the HA-hP2Y_2_R and VE-cadherin in HCAECs was observed by co-IP within 5 min of exposure to UTP, but was not detected 10 min later (Figure 2(a)). We also observed that treatment of HCAECs with UTP caused a transient association (maximum after 5 min) between VE-cadherin and VEGFR-2 (Figure 2(b)). Similar rapid and transient association between VEGFR-2 and HA-P2Y_2_R after UTP stimulation was observed by co-IP in a previous study performed in our laboratory [16]; therefore, we conclude that P2Y_2_R, VEGFR-2 and VE-cadherin form a transient complex upon P2Y_2_R activation. Since our previous studies indicated that the activated P2Y_2_R interacts with Src kinase and induces the Src-dependent transactivation of growth factor receptors, including VEGFR-2 [16], the epithelial growth factor receptor (EGFR), and platelet-derived growth factor receptor (PDGFR) [15], we hypothesized that Src activity is required for association between VE-cadherin and the P2Y_2_R. Accordingly, we found that pretreatment of HCAECs with the Src inhibitor, PP2, or with the VEGFR-2 inhibitor, SU1498, inhibited the ability of UTP to induce association between VE-cadherin and the P2Y_2_R (Figure 2(c)), suggesting that both Src and VEGFR-2 activity are required for recruitment of the P2Y_2_R to endothelial adherens junctions.

Previous studies have shown that VE-cadherin inhibits the internalization of VEGFR-2 in response to VEGF and, thus, VEGF-induced signaling [37]. Consistent with this finding, we found that over-expression of VE-cadherin in 1321N1 astrocytoma cells expressing the HA-hP2Y_2_R inhibited UTP-induced internalization of the HA-hP2Y_2_R (Supplemental Figure S1).

### 3.3. The P2Y_2_R Mediates Tyrosine Phosphorylation of VE-Cadherin

**Figure 3:**
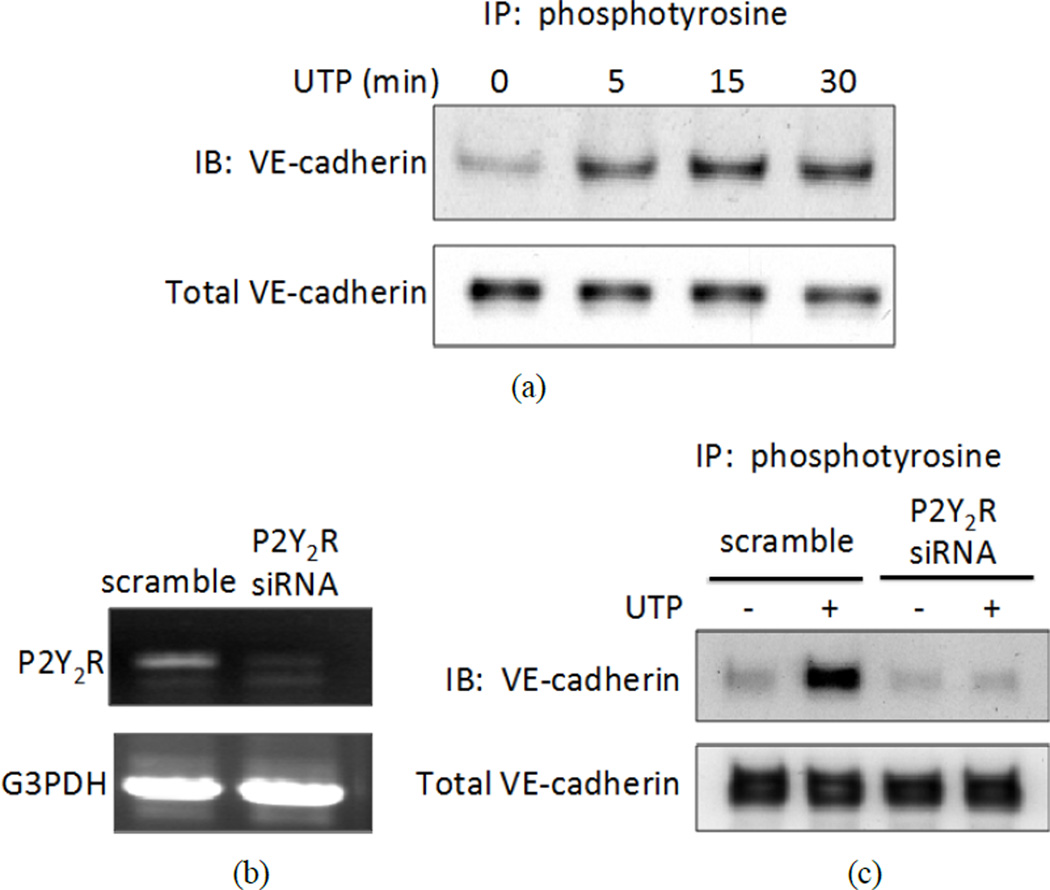
Activation of the P2Y2R by UTP causes sustained tyrosine phosphorylation of VE-cadherin. (a) HCAECs were treated with 100 µM UTP for the indicated time. Cell lysates were subjected to IP with anti-phosphotyrosine antibody followed by IB with anti-VE-cadherin antibody to detect tyrosine phosphorylated VE-cadherin. Cell lysates also were subjected to IB with anti-VE-cadherin antibody to detect total VE-cadherin in each sample; (b) and (c) HCAECs were transfected with either scrambled siRNA or P2Y2R-specific siRNA for 36 h; (b) Total RNA was extracted from cell lysates and RT-PCR was performed with either P2Y2R primers or G3PDH primers; (c) Transfected cells were incubated with or without 100 µMUTP for 5 min. Cell lysates were prepared and subjected to IP with anti-phosphotyrosine antibody and IB with anti-VE-cadherin antibody. The data shown are representative of results from 4 experiments.

Previous studies have shown that treatment of endothelial cells with VEGF causes interaction between VE-cadherin and VEGFR-2 [32] and that VEGF can stimulate the tyrosine phosphorylation of adherens junction proteins, including VE-cadherin, β-catenin, plakoglobin (γ-catenin), and p120 catenin [38]. Likewise, we found that the P2Y_2_R agonist UTP induced tyrosine phosphorylation of VE-cadherin in HCAECs that occurred within 5 min of treatment (Figure 3(a)) and was sustained for more than 30 min (not shown). To confirm that UTP-induced tyrosine phosphorylation is mediated by the P2Y_2_R, we suppressed expression of the endogenous P2Y_2_R in HCAECs with specific P2Y_2_R siRNA duplexes. Results indicated that down-regulation of P2Y_2_R mRNA expression inhibited UTP-induced tyrosine phosphorylation of VE-cadherin in HCAECs (Figures 3(b) and Figure 3(c)), demonstrating the involvement of the P2Y_2_R. In addition, expression of HA-hP2Y_2_R and VE-cadherin in P2 receptor-deficient 1321N1 astrocytoma cells enabled UTP to induce tyrosine phosphorylation of VE-cadherin (not shown), further supporting the ability of the P2Y_2_R to mediate VE-cadherin phosphorylation.

### 3.4. Src and VEGFR-2 Activity Are Required for P2Y_2_R-Mediated Tyrosine Phosphorylation of VE-Cadherin

**Figure 4:**
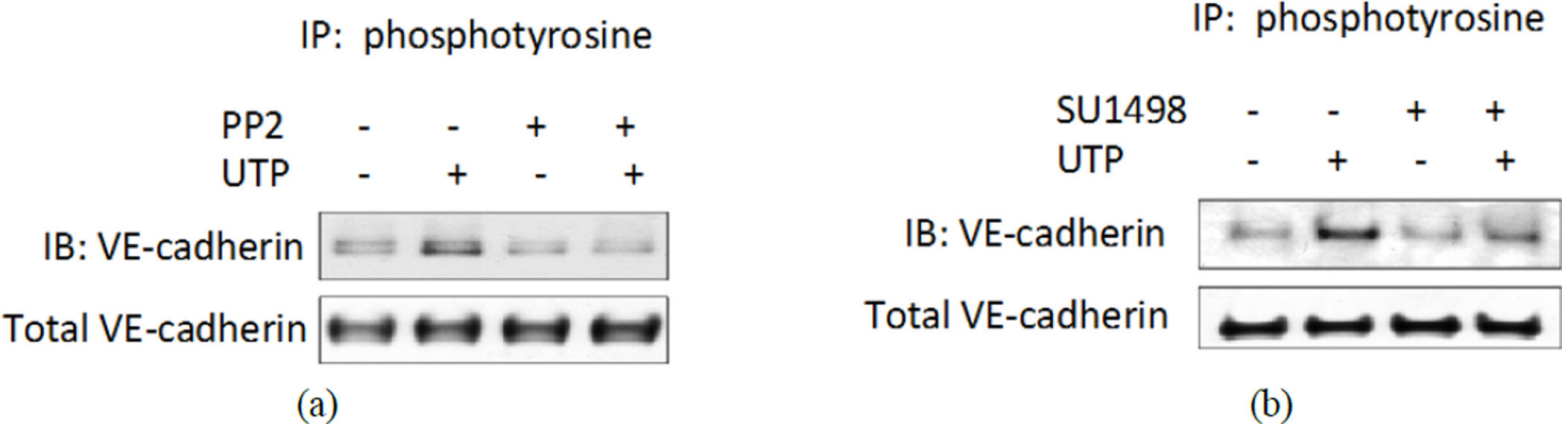
Src and VEGFR-2 kinase activity are necessary for P2Y2R-mediated tyrosine phosphorylation of VE-cadherin. (a) and (b) HCAECs expressing HA-tagged human P2Y2R were treated with the Src kinase inhibitor (1 µM PP2) or the VEGFR-2 kinase inhibitor (10 µM SU1498) for 30 min and then with 100 µM UTP for 5 min. Cell lysates were prepared and subjected to IP with anti-phosphotyrosine antibody and IB with anti-VE-cadherin antibody. Cell lysates also were subjected to IB with anti-VE-cadherin antibody to detect total VE-cadherin. Blots representative of 3 experiments are shown.

In endothelial cells, activation of the P2Y_2_R is known to cause a transient (peaking at 5 min) tyrosine phosphorylation of VEGFR-2, which leads to an increase in expression of the vascular cell adhesion molecule VCAM-1 that is dependent on the functional activity of Src and VEGFR-2 [16]. Here, we found that pretreatment of HCAECs with the Src kinase inhibitor PP2 or the VEGFR-2 kinase inhibitor SU1498 inhibited the UTP-induced interaction between HA-hP2Y_2_R and VE-cadherin (Figure 2(c)) as well as the UTP-induced tyrosine phosphorylation of VE-cadherin (Figure 4). The role of Src in mediating signal transduction between the P2Y_2_R and VE-cadherin was further demonstrated by using a mutant P2Y_2_R that lacks the C-terminal SH3-binding domains (Del-hP2Y_2_R) but functions identically to the wild type receptor with respect to calcium and ERK signaling. This deletion mutant was previously used to show that Src binds to the activated P2Y_2_R via the C-terminal SH3-binding domains [15] and that these domains are required for Src-dependent transactivation of VEGFR-2 and upregulation of VCAM-1 [16]. In this study, we found that UTP did not cause association between VE-cadherin and Del-hP2Y_2_R nor did UTP induce tyrosine phosphorylation of VE-cadherin in 1321N1 cells expressing DelhP2Y_2_R (Supplemental Figure S2). Together, these experiments suggest that binding of Src to SH3-binding domains in the P2Y_2_R is required for VEGFR-2 transactivation and the stimulation of VE-cadherin phosphorylation.

### 3.5. Both VE-Cadherin and VEGFR-2 Are Required for UTP-Induced Rac1 Activation

**Figure 5:**
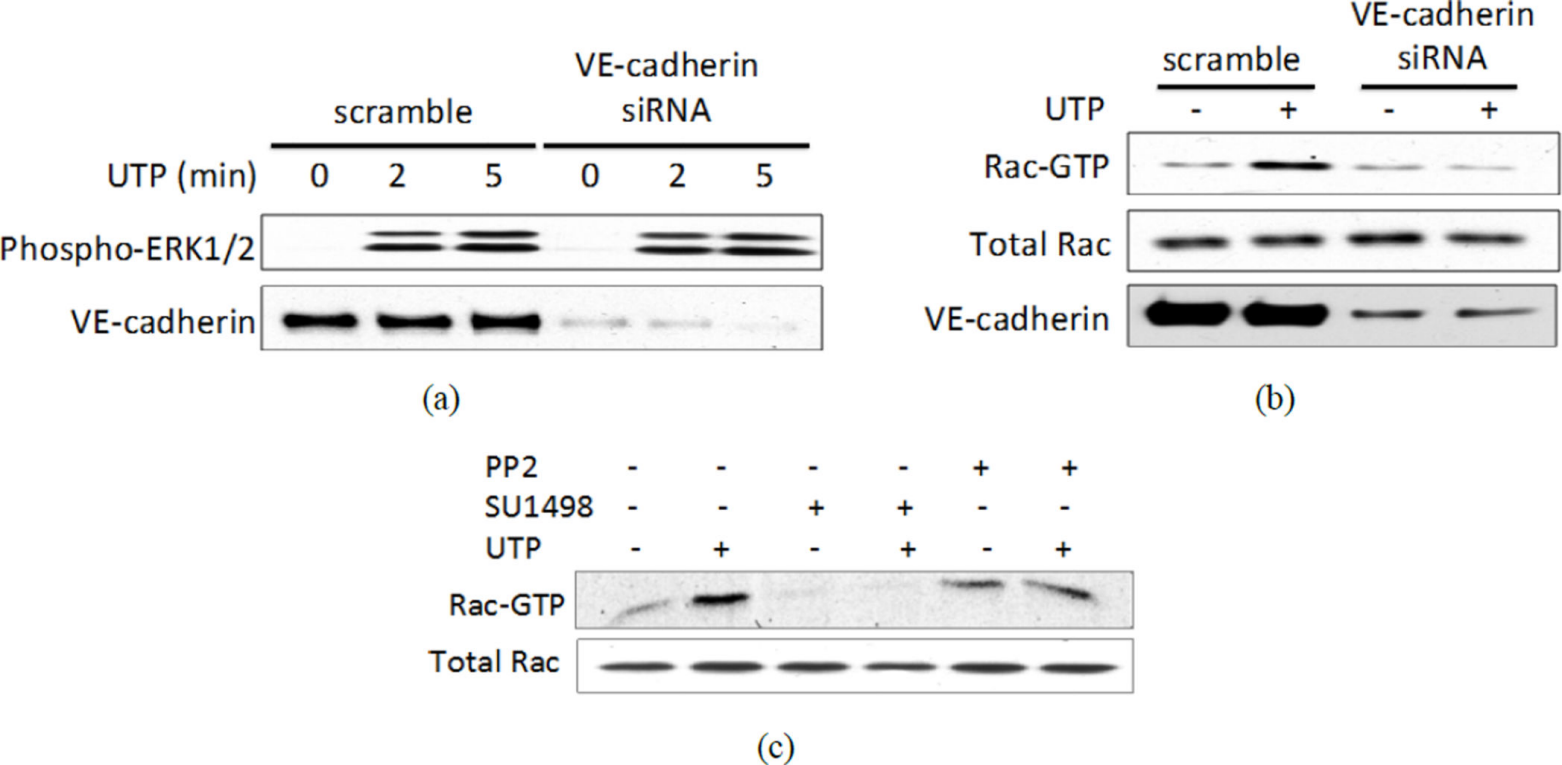
VE-cadherin expression is required for P2Y2R-mediated activation of Rac, but not ERK1/2. (a) and (b) HCAECs were transfected with either scrambled siRNA or VE-cadherin-specific siRNA for 36 h. (a) Transfected cells were incubated with 100 µM UTP for the indicated time and analyzed by IB with either anti-VE-cadherin antibody or anti-phospho-p42/44 (ERK1/2) antibody; (b) Transfected cells were subjected to a Rac activity assay, as described in the “Materials and Methods”; (c) HCAECs were treated as indicated with 1 µM PP2 or 10 µM SU1498 for 30 min, and then with 100 µM UTP for 5 min prior to measuring Rac activity. Cell lysates also were subjected to IB with anti-Rac antibody to detect total Rac. Blots representative of 3 experiments are shown.

Studies have demonstrated an important role for VE-cadherin-containing adherens junctions in regulating cell proliferation [39] and actin cytoskeletal organization [27]. To investigate the role of VE-cadherin in P2Y_2_R-mediated signal transduction, VE-cadherin-specific siRNA was used to down-regulate the expression of VE-cadherin in HCAECs (Figure 5(a)). It was reported that the presence of VE-cadherin decreased the phosphorylation of mitogen-activated protein kinase (MAPK) in response to VEGF [39]. We found that suppression of VE-cadherin expression did not inhibit UTP-induced activation of MAPK (*i.e.*, ERK1/2) (Figure 5(a)), but did inhibit UTP-induced activation of Rac1 (Figure 5(b)). Previous studies showed that Rac1 activation in HCAECs peaked after 5 min stimulation with 100 µM UTP [16]. UTP-induced Rac1 activation was inhibited by PP2 or SU1498 (Figure 5(c)), suggesting roles for Src and VEGFR-2 in the P2Y_2_R-mediated activation of Rac1. Consistent with a previous report [27], we found that knockdown of VE-cadherin expression in HCAECs caused an increase in basal Rho activity, which was not further stimulated by UTP (Supplemental Figure S3). Since both UTP-induced phosphorylation of VE-cadherin and activation of Rac1 are regulated by VEGFR-2, we conclude that VEGFR-2 activity is required for VE-cadherin-dependent activation of Rac1 mediated by the P2Y_2_R.

### 3.6. The P2Y_2_R Regulates p120 Catenin Phosphorylation via VEGFR-2 and VE-Cadherin

**Figure 6:**
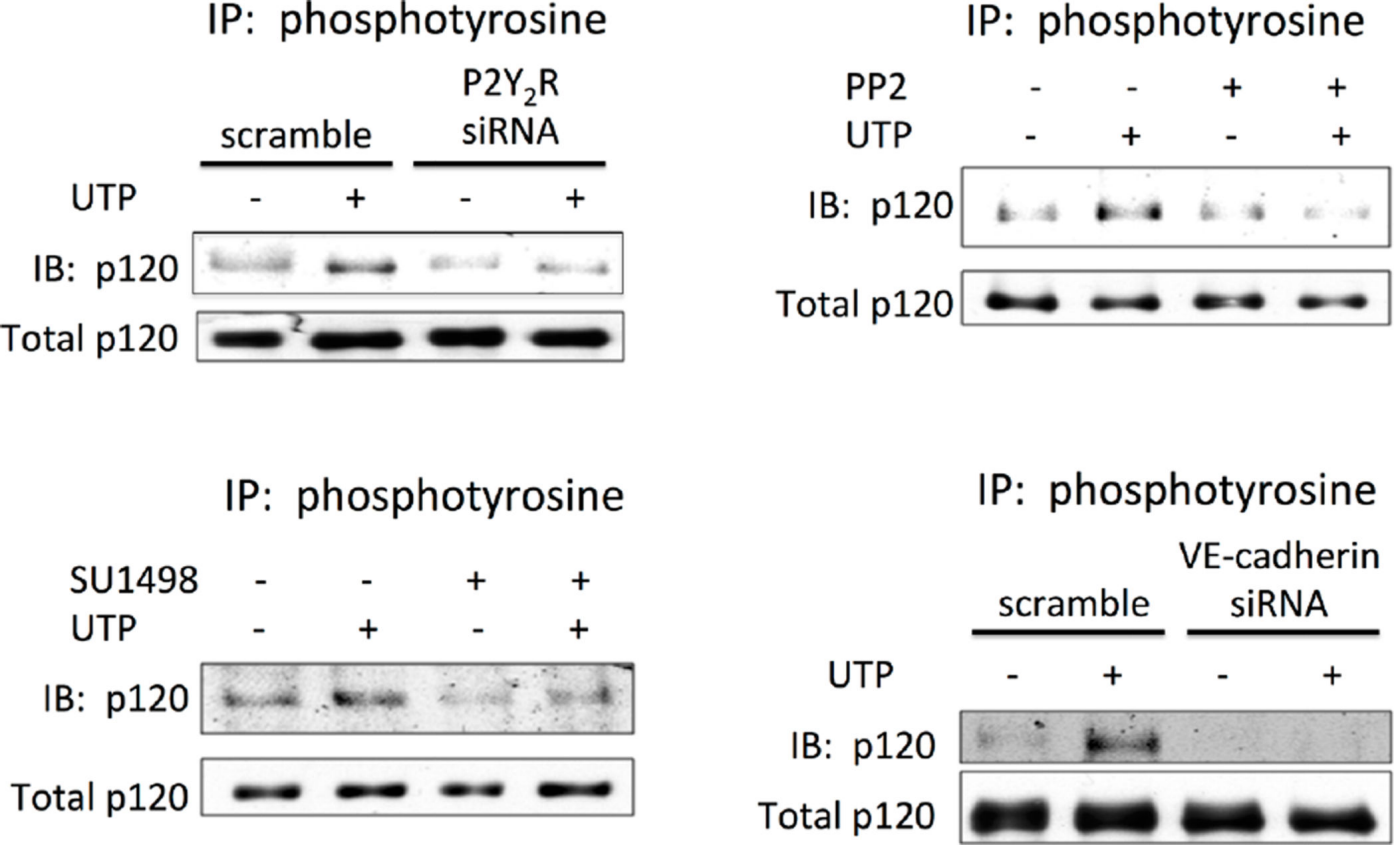
P2Y2R activation causes tyrosine phosphorylation of p120 catenin that is dependent upon Src and VEGFR-2 kinase activities and expression of VE-cadherin. (a) and (d) HCAECs were transfected with the indicated siRNA for 36 h and then incubated with or without 100 µM UTP for 5 min. (b) and (c) HCAECs were treated with 1 µM PP2 or 10 µM SU1498 for 30 min, and then incubated with or without 100 µM UTP for 5 min. (a)–(d), Cell lysates were prepared and subjected to IP with anti-phosphotyrosine antibody and IB with anti-p120 catenin antibody. Cell lysates were also subjected to IB with anti-p120 catenin antibody to detect total p120 catenin. Blots representative of 3 experiments are shown.

Other adherens junction proteins that interact with VE-cadherin provide a linkage with actin cytoskeleton as well as intracellular signaling pathways [28]. Among these adherens junction proteins, p120 catenin binds to the cytoplasmic domain of cadherins in the juxtamembrane region and regulates the activity of small Rho GTPases, thereby modulating actin cytoskeletal organization and cell motility [29]–[31]. Tyrosine phosphorylation of p120 catenin is dependent upon an interaction between p120 and VE-cadherin, thereby regulating endothelial barrier function [40]. We found that in HCAECs, UTP caused tyrosine phosphorylation of p120 catenin that was inhibited by transfection of cells with P2Y_2_R-specific siRNA, indicating that UTP-induced phosphorylation of p120 catenin is mediated by the P2Y_2_R (Figure 6(a)). UTP-induced tyrosine phosphorylation of p120 catenin was prevented by treatment of HCAECs with PP2 or SU1498 (Figures 6(b) and Figure 6(c)), indicating a role for Src-dependent VEGFR-2 transactivation in P2Y_2_R-mediated p120 catenin phosphorylation. Furthermore, downregulation of VE-cadherin with VE-cadherin-specific siRNA inhibited UTP-induced tyrosine phosphorylation of p120 catenin (Figure 6(d)), indicating that VE-cadherin is required for modulation of p120 catenin phosphorylation in response to P2Y_2_R activation.

### 3.7. p120 Catenin Regulates P2Y_2_R-Mediated Activation of Rac1

**Figure 7:**
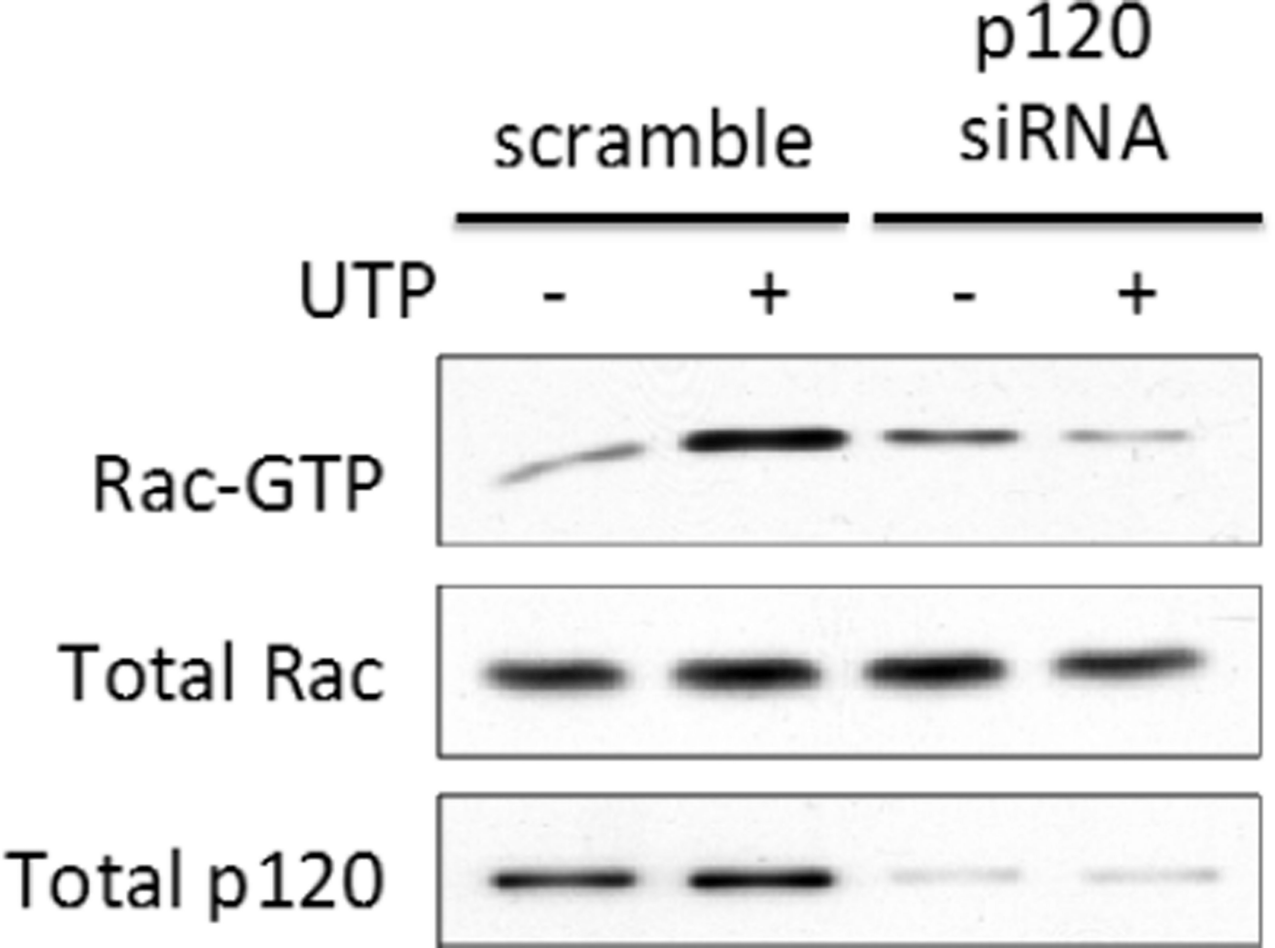
p120 catenin expression is required for P2Y2R-mediated activation of Rac1. HCAECs were transfected with either scrambled siRNA or p120 catenin-specific siRNA for 36 h and then incubated with or without 100 µM UTP for 5 min prior to measuring Rac1 activity. Cell lysates were prepared and subjected to IB with anti-Rac1 antibody or anti-p120 catenin antibody to detect total Rac1 and p120 catenin, respectively. Blots representative of 3 experiments are shown.

**Figure 8:**
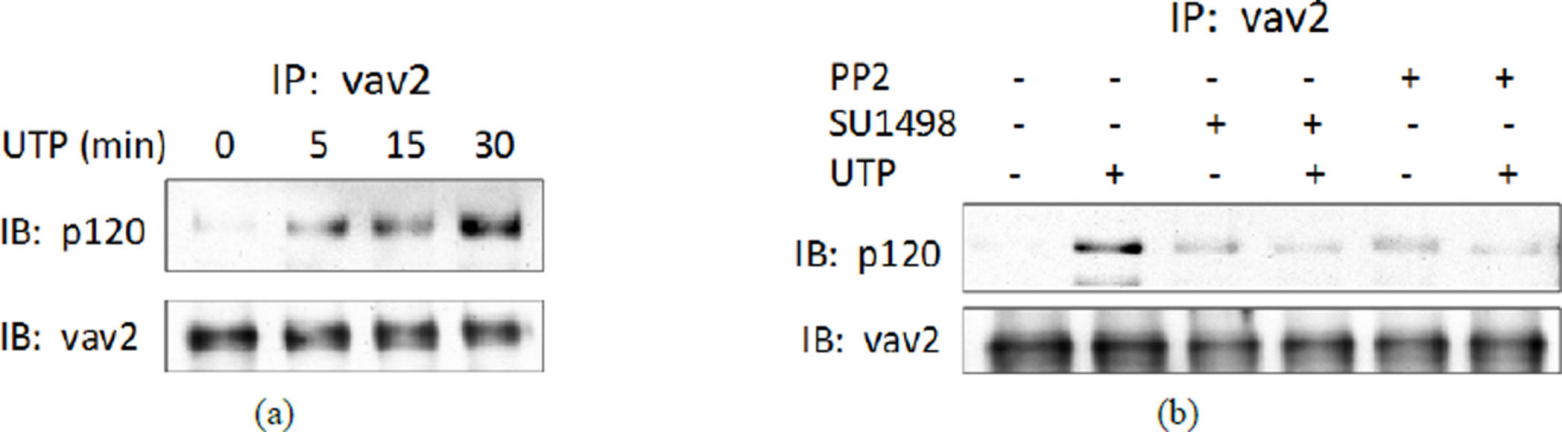
P2Y2R activation causes interaction between p120 catenin and vav2. (a) HCAECs were incubated with 100 µM UTP for the indicated times; (b) HCAECs were treated with 1 µM PP2 or 10 µM SU1498 for 30 min and then with 100 µM UTP for 5 min. (a) and (b) Cell lysates were prepared and subjected to IP with anti-vav2 antibody and IB with anti-p120 catenin or anti-vav2 antibody. Blots representative of 3 experiments are shown.

To examine the role of p120 catenin in VE-cadherin-dependent activation of Rac1 mediated by the P2Y_2_R, we transfected HCAECs with p120 catenin-specific siRNA to down-regulate expression of p120 catenin. UTP-induced activation of Rac1 was inhibited by p120 catenin-specific siRNA transfection (Figure 7), further supporting the idea that adherens junction proteins are necessary for the P2Y_2_R to modulate Rho GTPase activity. Transfection of HCAECs with p120 catenin-specific siRNA also increased the basal activity of Rho (not shown), similar to VE-cadherin-specific siRNA (Supplemental Figure S3). Previously, we found that vav2, a Rac GEF, regulated P2Y_2_R-mediated Rac1 activation, by demonstrating that expression of dominant negative vav2 inhibits Rac1 activity in UTP-treated 1321N1 cells expressing the P2Y_2_R [14]. It has been postulated that vav2 binding to p120 is required for P2Y_2_R-mediated Rac1 activation [29]. We found that UTP increased the interaction between p120 catenin and vav2 in HCAECs (Figure 8(a)), supporting the conclusion that vav2 interaction with p120 catenin is necessary for P2Y_2_R-mediated activation of Rac1. Furthermore, the UTP-induced interaction of vav2 and p120 catenin was inhibited by SU1498 or PP2 (Figure 8(b)), consistent with a role for Src-dependent VEGFR-2 activation in P2Y_2_R-mediated Rac1 activity.

## 4. Discussion

The endothelium plays an active role in vascular barrier function [41]–[45] and *in vivo* evidence indicates that the P2Y_2_R is an important regulator of microvascular permeability [5] and transendothelial passage of immune cells to injured or infected tissues [6]–[11]. Here, we examined the distribution of eGFP-tagged P2Y_2_Rs in quiescent and UTP-treated endothelial cells. In contrast to findings in migrating cells, where the activated P2Y_2_R is found evenly distributed on the cell surface [7] [13], we found that activation of the P2Y_2_R in confluent endothelial cell monolayers caused a rapid and transient translocation of eGFP-tagged P2Y_2_Rs to the cell-cell junctional zones. We also demonstrated that upon activation, the P2Y_2_R interacted transiently with VE-cadherin, a protein found specifically in adherens junctions of endothelial cells that is critical for maintaining the vascular barrier [46]. Based on these findings, we speculate that the endothelial P2Y_2_R affects vascular barrier function by relocating to endothelial cell junctions and interacting with VE-cadherin.

The role of VE-cadherin in controlling vascular integrity is well established [46] [47]. VE-cadherin functions as a dimer that forms homophilic interactions between neighboring endothelial cells and is linked by its cytoplasmic tail to other adherens junction proteins, such as p120, β-and γ-catenins (plakoglobin), and α-catenin through its interaction with β- or γ-catenins [28]. It is generally thought that VE-cadherin uses several mechanisms to control the vascular barrier, including serine and tyrosine phosphorylation, clathrin-dependent internalization and metalloprotease ADAM10-dependent shedding of VE-cadherin [1] [46] [48]–[50]. Also, a recent *in vivo* study showed that different tyrosine residues of VE-cadherin control leukocyte extravasation (Tyr731) and vascular permeability (Tyr685) and dephosphorylation of VE-cadherin at Tyr731 in cultured endothelial cells is triggered by leukocytes via the tyrosine phosphatase SHP-2, while the tyrosine phosphatase VE-PTP selectively affects phosphorylation of Tyr685 [51]. Although the dephosphorylation of specific tyrosine residues was not investigated in this study, we found that UTP treatment caused a sustained (>30 min) increase in overall tyrosine phosphorylation of VE-cadherin. Since the P2Y_2_R regulates both vascular permeability and leukocyte extravasation, it is likely that P2Y_2_R activation affects both Tyr685 and Tyr731, although this awaits further investigation. Likewise, both SHP-2 and VE-PTP are likely to be involved in controlling VE-cadherin tyrosine phosphorylation by the P2Y_2_R and the sustained increase in tyrosine phosphorylation of VE-cadherin induced by UTP in our endothelial cell culture experiments may be due to a lack of SHP-2 activity since leukocytes are required to trigger SHP-2 and the resulting dephosphorylation of Tyr731 [51]. In addition to causing tyrosine phosphorylation of VE-cadherin, we found that activation of P2Y_2_R in endothelial cells caused tyrosine phosphorylation of the VE-cadherin-associated protein, p120 catenin, interaction between the Rac GEF vav2 and p120 catenin and activation of Rac1, which seems to mimic the previously reported signaling pathway (*i.e*., Src-vav2-Rac-PAK) for VEGF-induced serine phosphorylation and β-arrestin-dependent endocytosis of VE-cadherin [52]. We, however, did not observe noticeable alterations in the distribution of VE-cadherin in HCAECs after 5 min or 30 min treatment of UTP, whereas VEGF was reported to induce rapid internalization of VE-cadherin within minutes [49], suggesting that the P2Y_2_R may not regulate endothelial barrier integrity via VE-cadherin endocytosis. Other potential mechanisms for the P2Y_2_R to regulate vascular integrity via VE-cadherin exist, but were not explored in this study. For example, P2Y_2_R activation has been reported to increase cytoplasmic calcium levels, activate ADAM10 metalloprotease [53], phosphorylate myosin light chain (MLC) [54] and focal adhesion kinase (FAK) [55], and upregulate VCAM-1 [56] and intercellular adhesion molecule-1 (ICAM-1) expression in vascular cells [16]. As mentioned above, VE-cadherin can be cleaved by ADAM10 in the ectodomain in response to calcium influx, while permeability to fluorescently labeled dextran and transendothelial T cell migration were noticeably decreased under ADAM10 inhibition [50]. Phosphorylation of MLC is critical for monocyteinduced tyrosine phosphorylation of VE-cadherin, and transendothelial monocyte migration [57] and FAK activity is required for VEGF-induced increase in vascular permeability and for β-catenin phosphorylation and dissociation from VE-cadherin [58]. Moreover, a role for VCAM-1 and ICAM-1 in VE-cadherin tyrosine phosphorylation has been demonstrated by using cross-linking antibodies to mimic the clustering of these adhesion molecules that occurs during transendothelial leukocyte migration [57] [59].

Likewise, the role of VE-cadherin in controlling cell proliferation is well recognized and requires interactions between VE-cadherin, Src kinases, catenins and the cell cytoskeleton [33] [60] [61]. VE-cadherin also regulates cell proliferation by preventing VEGF-induced internalization of VEGFR-2 that is required for activation of the mitogen-activated protein kinases ERK1/2 [37]. The P2Y_2_R in endothelial cells is known to promote cell proliferation via protein kinase C (PKC), phosphoinositide 3-kinase (PI3K) and ERK1/2 signaling [62]. Here, we found that over expression of VE-cadherin in cells stably expressing the HA-tagged P2Y_2_R inhibited UTP-induced internalization of the P2Y_2_R. Unlike Rac1 activation, however, UTP-induced ERK1/2 activation was unaffected by either down-regulation or over expression of VE-cadherin, suggesting that VE-cadherin is not involved in ERK1/2 signaling by the P2Y_2_R.

Interaction between P2Y_2_R and VE-cadherin induced by UTP was prevented by pretreatment of endothelial cells with PP2 or SU1498, compounds that inhibit Src and VEGFR-2 kinase activity, respectively. This suggests that Src and VEGFR-2 kinases regulate assembly of a VE-cadherin-associated complex with the P2Y_2_R that is important for downstream signaling to Rac1. In support of this idea, both Src and VEGFR-2 have been found to associate with the activated P2Y_2_R [15] [16] and we show that PP2 and SU1498 inhibited UTP-induced tyrosine phosphorylation of VE-cadherin and p120 catenin, interaction between p120 catenin and vav2, and activation of Rac1. Also, UTP-induced tyrosine phosphorylation of p120 catenin was dependent on VE-cadherin expression and UTP-induced Rac1 activation was dependent on VE-cadherin and p120 catenin expression, indicating that p120 catenin acts downstream of VE-cadherin to regulate Rac1 activity.

In addition to inducing VE-cadherin/P2Y_2_R interaction, UTP also caused a rapid and transient association between VEGFR-2 and VE-cadherin, suggesting that P2Y_2_R activation recruits VEGFR-2 to the VE-cadherin/ P2Y_2_R complex. VEGFR-2 is known to associate with VE-cadherin in response to not only VEGF stimulation but also shear stress in vascular endothelial cells [63]. Since nucleotides are released in response to mechanical stress [64], this suggests that the P2Y_2_R might act as a shear stress receptor in the vasculature to control VE-cadherin complexes and associated vascular barrier function.

Besides Src and VEGFR-2, integrins may also play a role in regulating P2Y_2_R/VE-cadherin interactions. It has been shown that fibronectin binding to integrins disrupts VE-cadherin-containing adherens junctions in a Src-dependent manner [65] and the P2Y_2_R contains an integrin-binding motif (RGD) that promotes interactions with the fibronectin-binding integrins, α_V_β_3_ and α_V_β_5_ [66]. We found that mutation of the RGD sequence in the P2Y_2_R to arginine-glycine-glutamate (RGE), prevents interaction between P2Y_2_R and α_V_β_3_/β_5_ integrins [66] and the RGE-P2Y_2_R mutant was unable to phosphorylate VE-cadherin in response to UTP (not shown), suggesting that P2Y_2_R/integrin interactions play a role in signaling to VE-cadherin. Since the G_q_-coupled P2Y_2_R has been reported to activate RhoA and Rac1 by coupling to G_o_ and G_12_ in an integrin-dependent manner [13] [14], it is likely that specific integrins and associated G proteins also regulate P2Y_2_R/VE-cadherin interactions.

Intracellular signaling transduced by VE-cadherin is complex and varies depending on whether the vasculature is growing (angiogenic) or established [47]. In confluent endothelial cultures, VE-cadherin activity more closely resembles an established, resting vascular bed, with VE-cadherin clustered in adherens junctions at cell-to-cell contacts and small GTPase activity favoring inhibition of RhoA activity, which decreases actomyosin contractility. We found that activation of the P2Y_2_R with UTP in confluent HCAEC cultures activates both RhoA and Rac1 and *in vivo* studies showed that the Rho-associated protein kinase inhibitor Y27632 blocks the increase in microvascular permeability induced by UTP [5]. Although the ultimate function of Rac1 activation by the P2Y_2_R in quiescent endothelial cells is unclear, it is known that Rac1 regulates the integrity of adherens junctions and participates in cytoskeletal rearrangements important for endothelial permeability induced by histamine, thrombin and VEGF [67] and transendothelial leukocyte migration [68]. Thus, Rac1 is likely to be involved in endothelial permeability and leukocyte recruitment that are also controlled by the P2Y_2_R. Interestingly, the expression of both dominant-negative and constitutively active forms of Rac1 increases the permeability of unstimulated endothelial cells [67], suggesting that precise control of Rac1 activity is needed to maintain the integrity of the vascular barrier.

## 5. Conclusion

**Figure 9:**
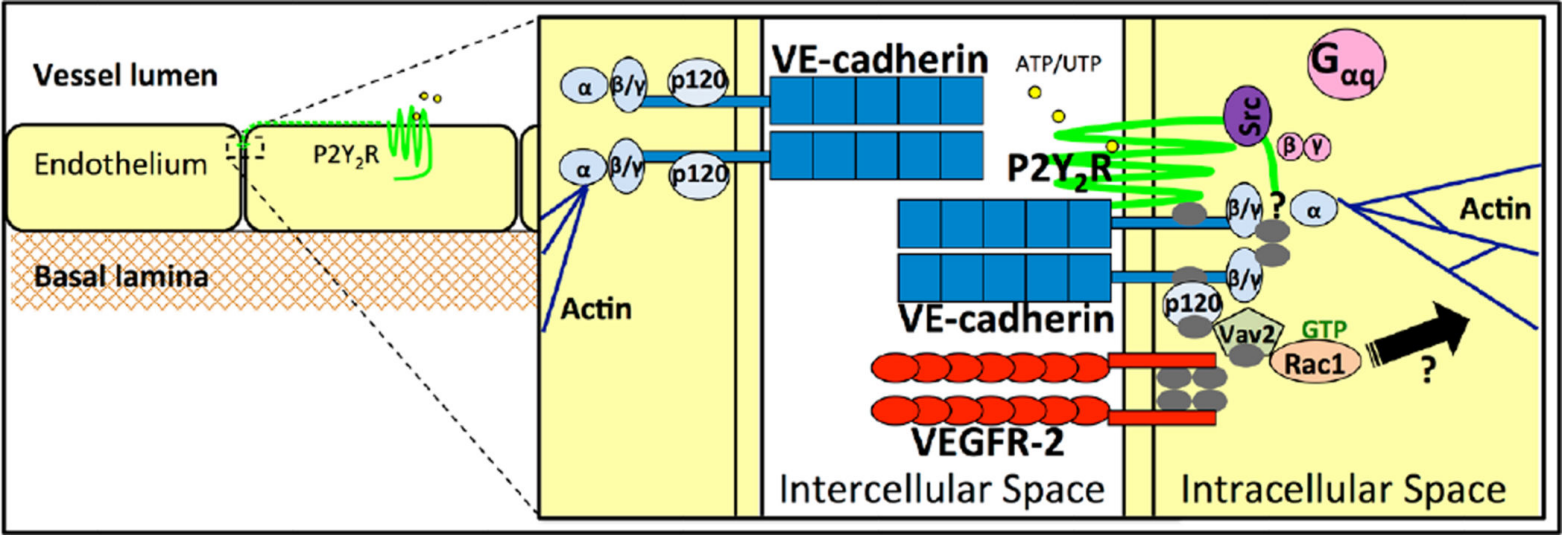
A schematic of the signaling pathways activated by the P2Y2R in endothelial cells that are thought to regulate vascular permeability. After activation by ATP or UTP, P2Y2R migrates to the cell-cell adherens junctions and transiently associates with VEGFR-2 and VE-cadherin, which are phosphorylated via a Src-dependent pathway. The kinase Src also phosphorylates VE-cadherin-bound p120 catenin, and possibly β- and γ-catenins as well, to further disrupt the adherens junctions. p120 then associates with the GEF vav2, activating Rac1, which is likely to induce cytoskeletal rearrangements to further facilitate the passage of macromolecules or leukocytes through the endothelial intercellular space. Grey ovals represent the phosphorylation sites, catenins are represented by light blue ovals and heterotrimeric G protein subunits are represented by pink circles.

Results presented here demonstrate that in endothelial cells, activation of the G protein-coupled P2Y_2_R induces the Src- and VEGFR-2-dependent activation of VE-cadherin in adherens junctions, which leads to the p120 catenin- and vav2-dependent activation of Rac1 (Figure 9). Since the P2Y_2_R can associate with VE-cadherin as well as VEGFR-2 [16] and α_V_β_3_/β_5_ integrins [66], these results suggest that the P2Y_2_R participates in a multi-receptor complex to regulate the function of adherens junctions. This study elucidates a novel mechanism whereby nucleotides act as signaling cues to modulate adherens junctions and activate Rac1 signaling in endothelium, an important process for regulating vascular permeability and responses to inflammation.

## Supplementary Material

01
