# Supplementary material for: The P2Y2 Receptor Interacts with VE-Cadherin and VEGF Receptor-2 to Regulate Rac1 Activity in Endothelial Cells
Source: J Biomed Sci Eng. Author manuscript; Available in PMC 2015 Feb 3. (PMC4314728; doi:10.4236/jbise.2014.714109)
Supplement: 01 [file NIHMS656366-supplement-01.pdf]

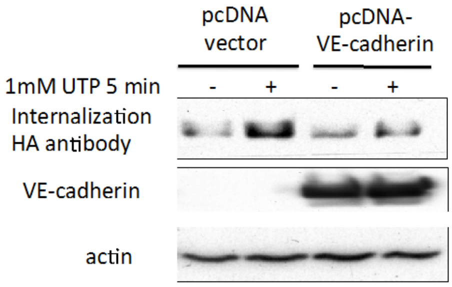

Supplemental Figure S1. VE-cadherin inhibits UTP-induced internalization of the HA-hP2Y$_2$R in 1321N1 astrocytoma cells. Human 1321N1 astrocytoma cells stably expressing the wild type HA-hP2Y$_2$R were transiently transfected with pcDNA3 or pcDNA3-VE-cadherin. Anti-HA antibodies were added to the medium and the cells were incubated with or without 1 mM UTP for 5 min to allow endocytosis to proceed. Surface-bound antibodies were removed from cells by acidic buffer wash and internalized anti-HA antibodies were detected by IB, as described in "Materials and Methods". Total cell lysates also were subjected to IB with anti-VE-cadherin or anti-actin antibodies. Blots representative of 3 experiments are shown.

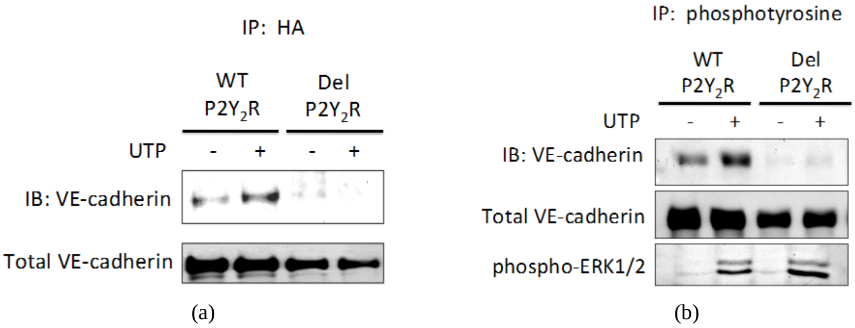

Supplemental Figure S2. The Src-binding domain of the P2Y$_2$R is required for the P2Y$_2$R to interact with and phosphorylate VE-cadherin. Human 1321N1 cells expressing the HA-tagged WT (wild type) or Del (deletion of prolines in the Src-binding domains) mutant hP2Y$_2$R were transfected with VE-cadherin cDNA in pcDNA3 and incubated with or without 100 µMUTP for 5 min. Cell lysates were prepared and subjected to IP with (a) anti-HA matrix or (b) anti-phosphotyrosine antibody, and IB with anti-VE-cadherin antibody. Cell lysates also were prepared and analyzed by immunoblotting with anti-VE-cadherin antibody. Blots representative of 3 experiments are shown.

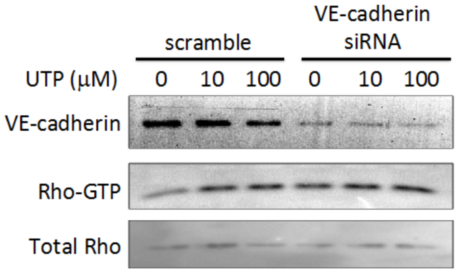

Supplemental Figure S3. Effect of down-regulation of VE-cadherin on UTP-induced activation of Rho in HCAECs. HCAECs were transfected with either scrambled or VE-cadherin-specific siRNA for 36 h, then incubated with the indicated concentration of UTP for 5 min prior to performing a Rho activity assay, as described in "Materials and Methods". Cell lysates also were prepared and analyzed by IB with anti-Rho antibody to detect total Rho in the samples. Blots representative of 3 experiments are shown.
